# Health-care access dimensions and ovarian cancer survival: SEER-Medicare analysis of the ORCHiD study

**DOI:** 10.1093/jncics/pkad011

**Published:** 2023-02-16

**Authors:** Mary Katherine Montes de Oca, Quan Chen, Elizabeth Howell, Lauren E Wilson, Clare Meernik, Rebecca A Previs, Bin Huang, Maria Pisu, Margaret I Liang, Kevin C Ward, Maria J Schymura, Andrew Berchuck, Tomi Akinyemiju

**Affiliations:** Department of Obstetrics and Gynecology, Duke University Medical Center, Durham, NC, USA; Division of Cancer Biostatistics and Kentucky Cancer Registry, University of Kentucky, Lexington, KY, USA; Department of Obstetrics and Gynecology, Duke University Medical Center, Durham, NC, USA; Department of Population Health Sciences, Duke University School of Medicine, Durham, NC, USA; Department of Population Health Sciences, Duke University School of Medicine, Durham, NC, USA; Division of Gynecologic Oncology, Duke Cancer Institute, Duke University School of Medicine, Durham, NC, USA; Division of Cancer Biostatistics and Kentucky Cancer Registry, University of Kentucky, Lexington, KY, USA; Division of Preventive Medicine and O’Neal Comprehensive Cancer Center, University of Alabama at Birmingham, Birmingham, AL, USA; Division of Gynecologic Oncology, Department of Obstetrics and Gynecology, Cedars-Sinai Medical Center, Los Angeles, CA, USA; Department of Epidemiology, Emory University, Atlanta, GA, USA; New York State Department of Health, New York State Cancer Registry, Albany, NY, USA; Division of Gynecologic Oncology, Duke Cancer Institute, Duke University School of Medicine, Durham, NC, USA; Department of Population Health Sciences, Duke University School of Medicine, Durham, NC, USA; Duke Cancer Institute, Duke University School of Medicine, Durham, NC, USA

## Abstract

**Background:**

Racial and ethnic disparities in ovarian cancer (OC) survival are well-documented. However, few studies have investigated how health-care access (HCA) contributes to these disparities.

**Methods:**

To evaluate the influence of HCA on OC mortality, we analyzed 2008-2015 Surveillance, Epidemiology, and End Results-Medicare data. Multivariable Cox proportional hazards regression models were used to estimate hazard ratios (HRs) and 95% confidence intervals (CIs) for the association between HCA dimensions (affordability, availability, accessibility) and OC-specific and all-cause mortality, adjusting for patient characteristics and treatment receipt.

**Results:**

The study cohort included 7590 OC patients: 454 (6.0%) Hispanic, 501 (6.6%) Non-Hispanic (NH) Black, and 6635 (87.4%) NH White. Higher affordability (HR = 0.90, 95% CI = 0.87 to 0.94), availability (HR = 0.95, 95% CI = 0.92 to 0.99), and accessibility scores (HR = 0.93, 95% CI = 0.87 to 0.99) were associated with lower risk of OC mortality after adjusting for demographic and clinical factors. Racial disparities were observed after additional adjustment for these HCA dimensions: NH Black patients experienced a 26% higher risk of OC mortality compared with NH White patients (HR = 1.26, 95% CI = 1.11 to 1.43) and a 45% higher risk among patients who survived at least 12 months (HR = 1.45, 95% CI = 1.16 to 1.81).

**Conclusions:**

HCA dimensions are statistically significantly associated with mortality after OC and explain some, but not all, of the observed racial disparity in survival of patients with OC. Although equalizing access to quality health care remains critical, research on other HCA dimensions is needed to determine additional factors contributing to disparate OC outcomes by race and ethnicity and advance the field toward health equity.

Racial and ethnic disparities in ovarian cancer (OC) outcomes persist and may be widening. Although non-Hispanic (NH) White women are more frequently diagnosed with OC compared with Black or Hispanic women in the United States (11.0 vs 9.1 vs 10.3 cases per 100 000, respectively) (1), NH White women survive longer than NH Black women after OC diagnosis, and the survival disparity between NH White and NH Black women is increasing (1-3). The current evidence suggests that disparities by race and ethnicity in receipt of guideline-concordant treatment account for some, but not all, of the survival disparity (4,5).

National Comprehensive Cancer Network (NCCN) guidelines for the treatment of OC recommend complete surgical staging and cytoreduction for all patients; observation for early-stage, low-grade disease; and systemic chemotherapy for advanced-stage, high-grade disease (6). However, receipt of guideline-concordant care differs by race and ethnicity, and these differences parallel the disparities observed in survival: Black women are less likely to receive cancer-directed surgery and chemotherapy concordant with NCCN guidelines (2,4,7-10), and Hispanic women are less likely to receive complete staging compared with Asian or Pacific Islander and White women (11).

The racial disparities in both treatment receipt and survival among Black and Hispanic patients highlight the importance of clarifying how health-care access (HCA) factors may contribute to these disparities. Five dimensions of HCA are described in the Penchansky and Thomas access framework: affordability (ability to pay), availability (service type, quality, and volume), accessibility (service geographic location), accommodation (service and resource organization), and acceptability (quality of patient–provider interaction, patient experience) (12). Prior studies of patients with OC have examined the association between individual components of HCA dimensions (eg, socioeconomic status [SES] and insurance status as indicators of affordability; facility volume as an indicator of availability) with guideline-concordant care and survival (11,13-15) and found that patients who were Black, low SES, or had poor access to care were least likely to receive guideline-concordant treatment (eg, Black vs White risk ratio = 0.75, 95% confidence interval [CI] = 0.66 to 0.84) and were at highest risk of death (eg, Black vs White risk ratio = 1.18, 95% CI = 1.11 to 1.26) (13). However, to our knowledge, no study has comprehensively evaluated multiple HCA dimensions simultaneously among a diverse sample of OC patients. In this retrospective cohort study, we evaluated the association between 3 HCA dimensions measurable in the Surveillance, Epidemiology, and End Results (SEER)-Medicare linked database (affordability, availability, and accessibility) and survival in Hispanic, NH Black, and NH White OC patients. We additionally evaluated the association between HCA dimensions and survival in a subcohort of patients who survived at least 12 months after diagnosis (ie, “treatment receipt subcohort” who survived long enough to receive primary treatment) and adjusted analyses for receipt of guideline-concordant treatment.

## Methods

### Study population

**Figure 1.**
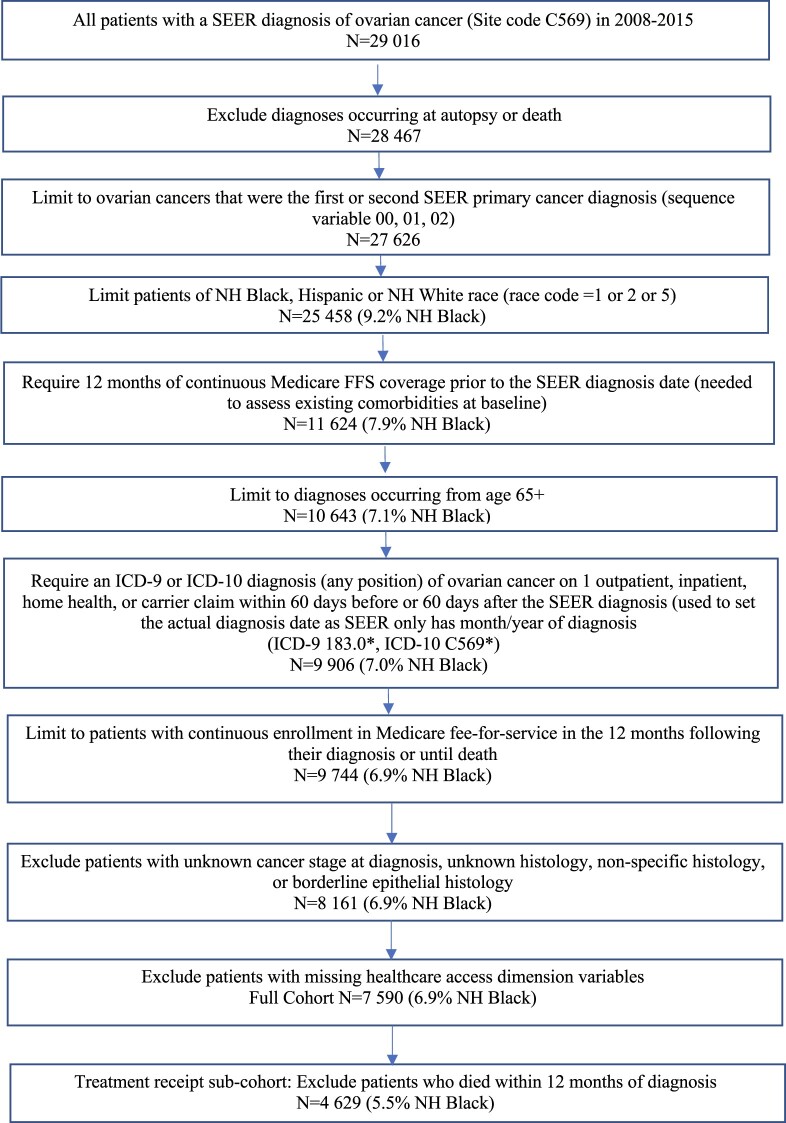
Participant flowchart for Hispanic, Non-Hispanic (NH) Black, and NH White ovarian cancer patients, Surveillance, Epidemiology, and End Results-Medicare 2008-2015. FFS = Fee For Service; ICD = International Classification of Diseases; SEER = Surveillance, Epidemiology, and End Results Program.

Hispanic, NH Black, and NH White women aged 65 years and older diagnosed with primary OC (SEER primary site code C569; epithelial, germ cell, or sex cord stromal histology) between 2008 and 2015 were identified from the SEER-Medicare linked dataset (Figure 1); the study population was limited to these racial and ethnic groups due to small numbers of patients with OC of other races and ethnicities in the database. The SEER-Medicare dataset provided data from 18 cancer registries in the United States linked with Medicare administrative claims data (16). Patients were required to have at least 12 months of continuous enrollment in Medicare fee-for-service parts A and B before diagnosis; at least 1 Medicare inpatient, outpatient, or carrier claim with a diagnosis code for OC (International Classification of Diseases [ICD]-9-CM and ICD-10-CM 183.0 or C569) within 2 months of the SEER diagnosis; and continuous fee-for-service Medicare enrollment in the 12 months following their diagnosis date or until death. Patients were excluded if they were diagnosed with borderline epithelial histology or fallopian tube or peritoneal cancers due to low case numbers. Patients were also excluded if they were missing data on cancer stage, grade, or histology at diagnosis (N = 1583) or missing variables used to create HCA dimension scores (N = 571); these exclusions did not differ by race and ethnicity.

For the treatment receipt subcohort, patients were additionally excluded if they died in the first year following diagnosis to allow for evaluation of disparities among patients who survived long enough to receive primary treatment. For all analyses of receipt of guideline-concordant systemic therapy, the cohort was limited to patients with a stage, grade, and histology combination at diagnosis for which NCCN clinical guidelines recommend systemic therapy. Decision trees for exclusions based on treatment guidelines are outlined in Supplementary Figures 1 and 2 (available online).

### SEER patient demographic and clinical characteristics

We obtained data on patient demographic and clinical characteristics from SEER, including race, ethnicity, age at diagnosis, stage, histology, marital status, geographic region of residence, and residence in a metropolitan area. We used validated coding algorithms to assess patient comorbidities and to calculate Charlson Comorbidity Index scores in the 12 months before OC diagnosis (17,18).

### All-cause and OC-specific mortality

All-cause and OC-specific mortality were assessed using date of death and cause of death data from SEER; this information was sourced from cancer registries and the National Death Index and was current through December 31, 2016. Study follow-up time was censored at the point when approximately 90% of the cohort had died or had reached the end of available study follow-up time, which occurred at 6 years (72 months) following diagnosis. In the full cohort, survival time for all-cause and OC-specific mortality was calculated from date of diagnosis to date of death or censoring; in the treatment receipt subcohort who survived at least 12 months, survival time was assessed starting 1 year after OC diagnosis date until death or censoring to assess disparities among patients who survived long enough to receive primary treatment.

### HCA dimension scores

A total of 35 patient-, census tract–, and regional-level variables (Supplementary Table 1, available online) measuring dimensions of health-care affordability (eg, census tract poverty rates, educational attainment), availability (eg, number of hospitals, specialists available per capita in region, consultation with a gynecologic oncologist), and accessibility (eg, residence in metropolitan vs rural area, distance traveled to care) were assessed. Confirmatory factor analysis (CFA) was used to identify the most influential variables for each dimension of HCA and create composite scores of affordability, availability, and accessibility at the patient level. Factor loadings from CFA were used to generate HCA dimension scores ranging from −3 to 4, with lower values representing lower access for the dimension. For instance, lower affordability scores correspond to being dual enrolled in Medicaid and Medicare or living in a census tract with greater poverty; lower availability scores correspond to lower average hospital quality or fewer physicians and hospitals in the patient’s region of residence; and lower accessibility scores correspond to a patient living in a rural area or living further from health care (Supplementary Table 1, available online). CFA for HCA dimensions is described in Supplementary Table 2, Supplementary Methods (available online), and in a prior publication (19).

### Receipt of guideline-concordant surgery

Patients were considered to have received OC-directed surgery if they had a Medicare claim that indicated an ovarian surgical procedure in the 2 months before or 6 months post diagnosis or had OC primary site surgery documented in the SEER registry data. Receipt of guideline-concordant surgery was based on NCCN clinical guidelines (Supplementary Figures 1 and 2, available online). We considered the gold standard of guideline-concordant surgery to be receipt of comprehensive staging laparotomy, including total abdominal hysterectomy (if no prior hysterectomy), bilateral salpingo-oophorectomy, lymph node dissection (if applicable), omentectomy, or pelvic or para-aortic lymph node biopsy (for Stage I-IIIB). Receipt of specific procedures in administrative claims data was difficult to accurately assess. Therefore, we selected a subset of surgical procedure codes, Current Procedural Terminology codes, and SEER registry primary site surgical codes that reliably represented receipt of guideline-concordant OC surgery (Supplementary Table 3, available online). Patients without relevant claims and without SEER documentation of a surgery that met guidelines were considered to have not received guideline-concordant surgery.

### Initiation and completion of guideline-concordant systemic therapy

NCCN Clinical Practice Guidelines were used (Supplementary Figures 1 and 2, available online) to determine recommended systemic therapy by tumor characteristics (20). We examined 2 systemic therapy outcomes: initiation of a recommended treatment regimen and completion of the recommended cycles of treatment. Patients were considered to have initiated guideline-concordant systemic therapy if they had at least 1 Medicare claim for administration of recommended systemic therapies according to the patient’s stage, grade, and histology (Supplementary Figures 1 and 2; Supplementary Table 4, available online) in the 12 months following the diagnosis date (20). Patients were considered to have completed the recommended number of systemic therapy cycles if they had the recommended number of therapy administration claims spaced at least 20 days apart in the 12 months following diagnosis.

### Statistical analysis

The distribution of patient demographic and clinical characteristics, HCA scores, and treatment receipt were described overall and stratified by patient race. Group-level differences were tested using Kruskal-Wallis and Cochran-Mantel-Haenszel tests as appropriate. In the full cohort, univariate- and multivariable-adjusted Cox proportional hazards regression models were used to estimate hazard ratios (HRs) and 95% confidence intervals for race and ethnicity and HCA scores with all-cause mortality and OC-specific mortality. Kaplan-Meier plots, Schoenfeld residual plots, and inclusion of time-varying covariates in the models were used to assess potential violations of the proportional hazard assumption; there was no evidence of violation of the proportional hazard assumption for our main covariates of interest. Cause-specific hazards were calculated for OC-specific mortality to account for competing risks of death from other causes. In the treatment receipt subcohort, estimates for race and ethnicity and HCA were further adjusted for guideline-concordant care, consultation with a gynecologic oncologist, and distance in miles to the patient’s surgical facility. All multivariable models were adjusted for age at diagnosis (65-70 years, 71-75 years, 76-80 years, 81+ years), stage at diagnosis (I, II, III, IV), tumor histology (Type I epithelial, Type II epithelial, other), US Census region of residence (Northeast, South, Midwest, West, other), and presence of individual comorbid conditions (yes, no). Disparities between NH Black and NH White survival rates attributable to key covariates were calculated using the percent reduction in hazard ratio estimates (excess risk) explained by each measure compared with a base model including patient demographics, tumor characteristics, and comorbid conditions, though this method does not account for the precision of estimates (21). Models with interaction terms between patient race and ethnicity and each HCA dimension were used to evaluate potential interaction between race and ethnicity and HCA dimensions on mortality.

## Results

### Study population and clinical characteristics

The full study cohort included 7590 patients with OC diagnosed from 2008 to 2015: 454 (6.0%) Hispanic, 501 (6.6%) NH Black, and 6635 (87.4%) NH White (Table 1). NH Black patients were more likely to have been diagnosed with stage IV cancer than NH White or Hispanic patients (44% vs 36% and 40%, respectively). NH Black and Hispanic patients had higher average comorbidity burden at OC diagnosis than NH White patients (Mean Charlson Comorbidity Index: 3.4 and 3.1, respectively, vs 2.4). Additionally, NH Black and Hispanic patients had lower HCA scores for affordability (−0.7 and −0.4 vs 0.1) and availability (−0.1 and −0.1 vs 0.0) than NH White patients, and higher scores for accessibility (0.1 and 0.2 vs 0.0). Sixty-one percent of patients in the full cohort survived at least 1 year following their OC diagnosis.

**Table 1.** Patient demographic, clinical, and health-care access characteristics for OC patients in the full (n = 7590) and treatment receipt subcohorts (n = 4629), SEER-Medicare 2008-2015^a^

	Full cohort			Treatment subcohort (survived ≥12 months)		
	Non-Hispanic White	Non-Hispanic-Black	Hispanic	Non-Hispanic White	Non-Hispanic Black	Hispanic
	6635	501	454	4105	255	269
Age at OC diagnosis, mean (SD)	76.9 (7.1)	75.9 (6.5)	75.9 (6.7)	75.3(6.3)	74.8 (5.8)	74.2 (6.1)
Affordability score, mean (SD)	0.1 (0.9)	−0.7 (0.8)	−0.4 (1.0)	0.2 (0.9)	−0.7 (0.8)	−0.3 (1.0)
Availability score, mean (SD)	0.0 (0.9)	−0.1 (0.9)	−0.1 (0.9)	0.0 (0.9)	−0.1 (0.8)	−0.1 (0.9)
Accessibility score, mean (SD)	0.0 (0.5)	0.1 (0.4)	0.2 (0.4)	0.0 (0.5)	0.1 (0.4)	0.1 (0.4)
Comorbidity score, mean (SD)	2.4 (2.0)	3.4 (2.6)	3.1 (2.6)	2.1 (1.8)	3.1 (2.3)	2.4 (2.1)
Stage at diagnosis, No. (%)						
I	881 (13.3)	61 (12.2)	56 (12.3)	448 (10.9)	22 (8.6)	27 (10)
II	504 (7.6)	35 (7)	31 (6.8)	423 (10.3)	24 (9.4)	29 (10.8)
III	2842 (42.8)	182 (36.3)	186 (41)	2068 (50.4)	127 (49.8)	123 (45.7)
IV	2408 (36.3)	223 (44.5)	181 (39.9)	1166 (28.4)	82 (32.2)	90 (33.5)
Histology, No. (%)						
Type I epithelial	831 (12.5)	61 (12.2)	>65	443 (10.8)	<40	<40
Type II epithelial	5751 (86.7)	425 (84.8)	375 (82.6)	3644 (88.8)	218 (85.5)	232 (86.2)
Other	53 (0.8)	15 (3.0)	<11	18 (0.4)	<11	<11
Married, No. (%)	2951 (44.5)	108 (21.6)	163 (35.9)	2080 (50.7)	60 (23.5)	103 (38.3)
Lives in metropolitan area, No. (%)	5572 (84.0)	446 (89.0)	416 (91.6)	3462 (84.3)	231 (90.6)	247 (91.8)
Lives in rural area, No. (%)	143 (2.2)	<11	<11	81 (2)	<11	<11
Geographic region, No. (%)						
Midwest	851 (12.8)	75 (15)	<20	486 (11.8)	34 (13.3)	<20
Other	584 (8.8)	61 (12.2)	<11	349 (8.5)	26 (10.2)	<11
Northeast	1458 (22)	112 (22.4)	70 (15.4)	889 (21.7)	66 (25.9)	43 (16)
South	1007 (15.2)	161 (32.1)	16 (3.5)	589 (14.3)	80 (31.4)	<11
West	2735 (41.2)	92 (18.4)	345 (76)	1792 (43.7)	49 (19.2)	203 (75.5)
Dual enrolled in Medicaid, No. (%)	668 (10.1)	192 (38.3)	210 (46.3)	301 (7.3)	82 (32.2)	109 (40.5)
Myocardial infarction, No. (%)	172 (2.6)	17 (3.4)	13 (2.9)	74 (1.8)	<11	<11
Hypertension, No. (%)	4277 (64.5)	425 (84.8)	303 (66.7)	2505 (61)	215 (84.3)	168 (62.5)
Peripheral vascular disease, No. (%)	589 (8.9)	67 (13.4)	45 (9.9)	263 (6.4)	24 (9.4)	17 (6.3)
Congestive heart failure, No. (%)	579 (8.7)	79 (15.8)	58 (12.8)	225 (5.5)	31 (12.2)	22 (8.2)
Dementia, No. (%)	102 (1.5)	18 (3.6)	15 (3.3)	27(0.7)	<11	<11
Cerebrovascular disease, No. (%)	537 (8.1)	41 (8.2)	35 (7.7)	275 (6.7)	17 (6.7)	11 (4.1)
Chronic obstructive pulmonary disease, No. (%)	991 (14.9)	83 (16.6)	70 (15.4)	545 (13.3)	42 (16.5)	28 (10.4)
Rheumatologic disease, No. (%)	250 (3.8)	22 (4.4)	21 (4.6)	157 (3.8)	14 (5.5)	14 (5.2)
Peptic ulcer disease, No. (%)	89 (1.3)	<11	<11	43(1)	<11	<11
Mild liver disease, No. (%)	248 (3.7)	30 (6)	38 (8.4)	130 (3.2)	12 (4.7)	15 (5.6)
End stage renal disease, No. (%)	446 (6.7)	66 (13.2)	42 (9.3)	195 (4.8)	26 (10.2)	11 (4.1)
Diabetes, No. (%)	1265 (19.1)	185 (36.9)	163 (35.9)	646 (15.7)	95 (37.3)	78 (29)
Diabetes with complications, No. (%)	282 (4.3)	54 (10.8)	49 (10.8)	111 (2.7)	27 (10.6)	22 (8.2)
Hemiplegia or paraplegia, No. (%)	32 (0.5)	<11	<11	<11	<11	<11
Moderate or severe liver disease, No. (%)	14 (0.2)	<11	<11	<11	<11	<11
Year of diagnosis, No. (%)						
2008	976 (14.7)	73 (14.6)	67 (14.8)	590 (14.4)	38 (14.9)	37 (13.8)
2009	884 (13.3)	64 (12.8)	48 (10.6)	554 (13.5)	34 (13.3)	28 (10.4)
2010	849 (12.8)	54 (10.8)	59 (13)	508 (12.4)	25 (9.8)	35 (13)
2011	770 (11.6)	75 (15)	53 (11.7)	471 (11.5)	36 (14.1)	35 (13)
2012	780 (11.8)	69 (13.8)	55 (12.1)	487 (11.9)	33 (12.9)	26 (9.7)
2013	791 (11.9)	68 (13.6)	53 (11.7)	483 (11.8)	40 (15.7)	27 (10)
2014	783 (11.8)	54 (10.8)	64 (14.1)	492 (12)	28 (11)	41 (15.2)
2015	802 (12.1)	44 (8.8)	55 (12.1)	520 (12.7)	21 (8.2)	40 (14.9)
Surgery met guideline recommendations, No. (%)	2570 (38.7)	143 (28.5)	168 (37)	2053 (50.0)	102 (40.0)	137 (50.9)
Initiated recommended systemic therapy, No. (%)	4582 (69.1)	306 (61.1)	312 (68.7)	3581 (87.2)	214 (84.0)	235 (87.4)

a OC = ovarian cancer; SEER = Surveillance, Epidemiology, and End Results Program.

### Treatment receipt subcohort surviving at least 12 months following diagnosis

**Figure 2.**
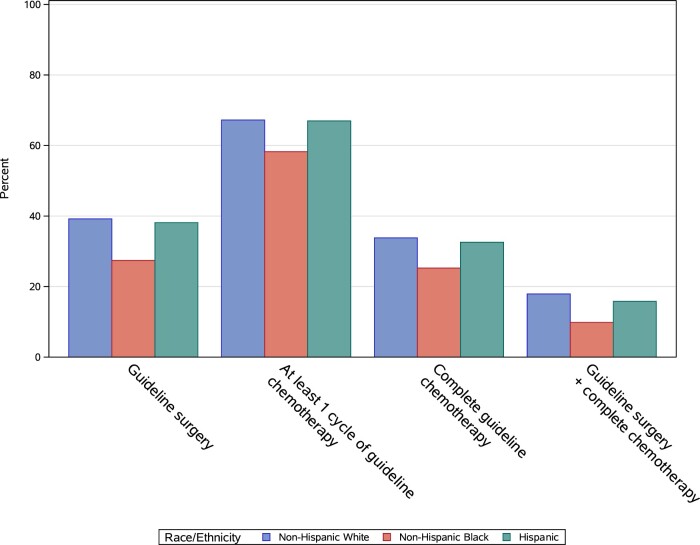
Treatment receipt among patients surviving at least 12 months after ovarian cancer (OC) diagnosis (N = 4629).

In the subcohort of patients who survived at least 1 year following OC diagnosis, 60% of NH Black patients did not receive surgery that met guidelines based on their tumor stage, histology, and grade compared with 50% of NH White patients and 49% of Hispanic patients (Figure 2). Eighty-eight percent of patients surviving 12 months initiated the recommended course of systemic therapy, with no differences observed by patient race and ethnicity. However, NH Black patients were less likely to complete the recommended number of cycles of systemic therapy (Figure 2; 42% of NH Black vs 49% and 50% of NH White and Hispanic patients, respectively).

### HCA and all-cause mortality in the full cohort

**Figure 3.**
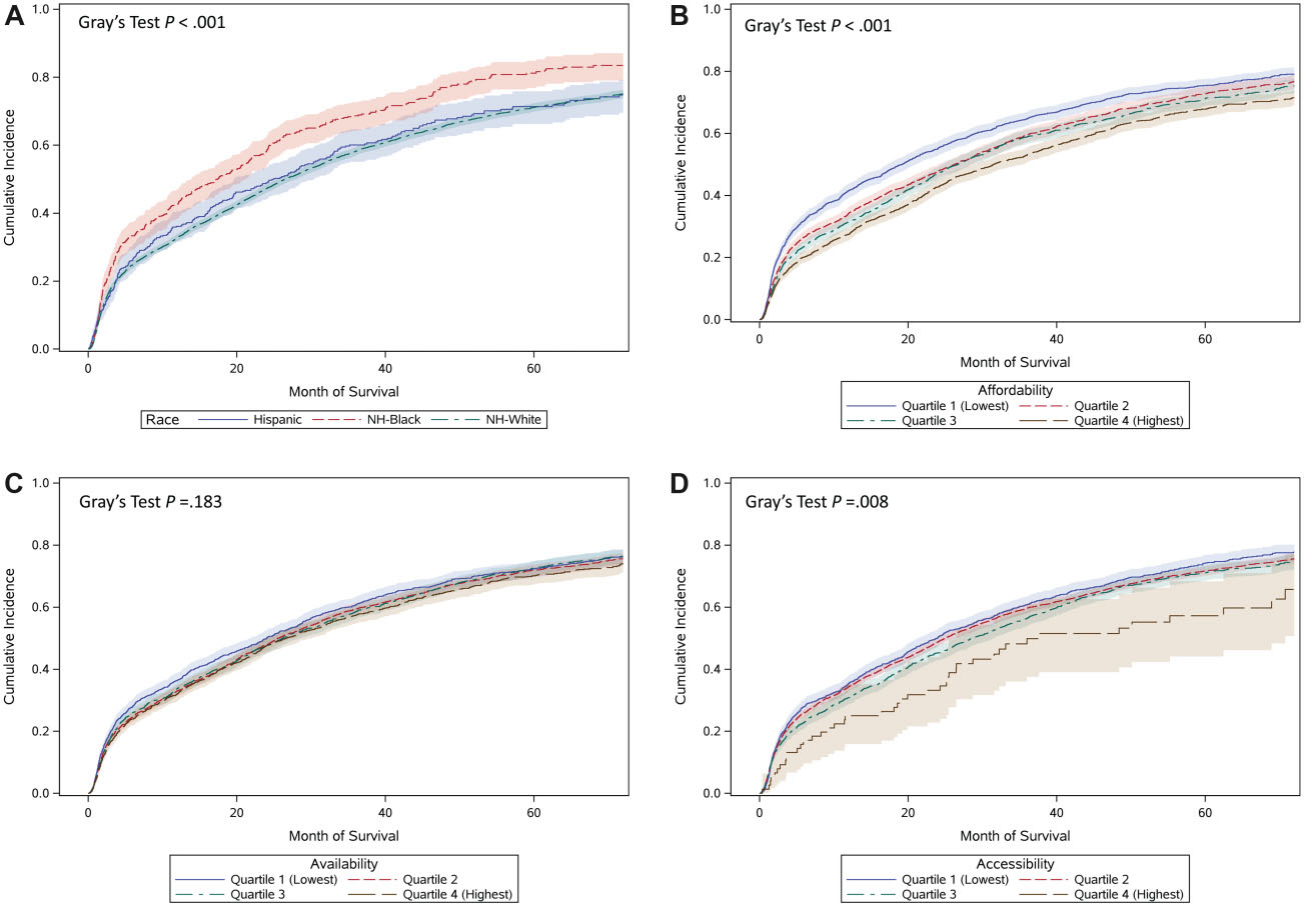
**A)** Cumulative incidence functions for ovarian cancer mortality in the full cohort stratified by Hispanic, non-Hispanic (NH) Black, and NH White race and ethnicity. **B)** Cumulative incidence functions for ovarian cancer mortality in the full cohort stratified by quartiles of Affordability score. **C)** Cumulative incidence functions for ovarian cancer mortality in the full cohort stratified by quartiles of Availability score. **D)** Cumulative incidence functions for ovarian cancer mortality in the full cohort stratified by quartiles of Accessibility score.

Median follow-up time was 21.3 months (interquartile range = 5.6 to 43.6); follow-up was censored at 72 months (6 years) post diagnosis when approximately 90% of the cohort had died or reached the end of available follow-up time. Cumulative incidence of death from OC was 63.7% (95% CI = 62.3% to 65.1%) at 6 years post diagnosis (Figure 3); NH Black patients had higher cumulative incidence of OC death at 1, 3, 5, and 6 years post diagnosis than NH White and Hispanic patients (log-rank *P* < .001). In multivariable adjusted Cox regression, NH Black patients showed increased risk of all-cause mortality in the full cohort (HR = 1.15, 95% CI = 1.03 to 1.29) and among patients diagnosed with advanced-stage cancer (HR = 1.25, 95% CI = 1.08 to 1.46) compared with NH White patients. Higher affordability scores were associated with lower risk of death from any cause among the full cohort (HR = 0.92, 95% CI = 0.89 to 0.95) and among patients with advanced-stage cancer (HR = 0.91, 95% CI = 0.87 to 0.96) compared with patients with lower scores (Table 2). Hispanic patients had similar rates of all-cause mortality as NH White patients in the full cohort and among patients with advanced stage disease.

**Table 2.** Association of race and ethnicity and HCA dimensions with risk of all-cause mortality (HR and 95% confidence intervals) in the full cohort (N = 7590), advanced-stage disease (N = 2812), and treatment receipt subcohort surviving at least 1 year (N = 4629)

	Patient characteristics^a^	Patient characteristics + affordability score^b^	Patient characteristics + availability score^c^	Patient characteristics + accessibility score^d^	Patient characteristics + 3 HCA scores	Estimated percentage of Non-Hispanic Black/Non-Hispanic White survival disparity explained
Full cohort						
Affordability score		0.92 (0.89 to 0.95)			0.92 (0.89 to 0.96)	31.8%
Availability score			0.98 (0.94 to 1.02)		1.00 (0.96 to 1.04)	0%
Accessibility score				0.93 (0.88 to 0.98)	0.97 (0.91 to 1.02)	−4.5%
Race (ref Non-Hispanic White)						
Hispanic	1.01 (0.90 to 1.14)	0.96 (0.85 to 1.09)	1.01 (0.90 to 1.14)	1.02 (0.90 to 1.15)	0.97 (0.86 to 1.10)	
Non-Hispanic Black	1.22 (1.09 to 1.36)	1.15 (1.03 to 1.29)	1.22 (1.09 to 1.36)	1.23 (1.10 to 1.37)	1.16 (1.04 to 1.30)	
Advanced stage at diagnosis (stage IV)						
Affordability score		0.91 (0.87 to 0.96)			0.91 (0.87 to 0.96)	21.8%
Availability score			0.98 (0.93 to 1.04)	1.00 (0.94 to 1.06)		0%
Accessibility score				0.98 (0.89 to 1.07)	0.99 (0.89 to 1.10)	−6.2%
Race (ref NH White)						
Hispanic	1.00 (0.84 to 1.18)	0.95 (0.80 to 1.13)	1.00 (0.84 to 1.18)	1.01 (0.85 to 1.19)	0.96 (0.80 to 1.14)	
NH Black	1.32 (1.14 to 1.53)	1.25 (1.08 to 1.46)	1.32 (1.14 to 1.53)	1.34 (1.15 to 1.55)	1.26 (1.08 to 1.47)	
Survived at least 1 year post diagnosis^e^						
Affordability score		0.97 (0.92 to 1.03)			0.98 (0.93 to 1.04)	12.0%
Availability score			0.99 (0.93 to 1.06)		0.99 (0.93 to 1.06)	0%
Accessibility score				0.94 (0.86 to 1.03)	0.95 (0.86 to 1.04)	−4.0%
Race (ref NH White)						
Hispanic	0.86 (0.70 to 1.06)	0.85 (0.69 to 1.05)	0.86 (0.7 to 1.06)	0.87 (0.70 to 1.07)	0.86 (0.69 to 1.06)	
NH Black	1.25 (1.02 to 1.52)	1.22 (1.00 to 1.49)	1.25 (1.02 to 1.52)	1.26 (1.03 to 1.53)	1.24 (1.01 to 1.52)	
Consulted gynecologic oncologist	0.93 (0.83 to 1.03)	0.93 (0.83 to 1.04)	0.93 (0.83 to 1.03)	0.93 (0.83 to 1.03)	0.93 (0.83 to 1.04)	
Distance to treatment facility, miles	1.00 (1.00 to 1.00)	1.00 (1.00 to 1.00)	1.00 (1.00 to 1.00)	1.00 (1.00 to 1.00)	1.00 (1.00 to 1.00)	
Surgery met guidelines (ref No)	0.90 (0.81 to 0.99)	0.90 (0.82 to 0.99)	0.90 (0.81 to 0.99)	0.90 (0.82 to 0.99)	0.90 (0.82 to 1.00)	7.0% (all treatment variables)^f^
Completed recommended cycles of chemotherapy (ref No)	0.89 (0.81 to 0.98)	0.89 (0.81 to 0.98)	0.89 (0.81 to 0.98)	0.90 (0.81 to 0.98)	0.89 (0.81 to 0.98)	

a Patient age at diagnosis, stage at diagnosis, tumor histology, region of residence, and comorbid conditions. HCA = health-care access; HR = hazard ratio; NH = Non-Hispanic.

b Affordability score: weighted factor score of measures indicating patient’s ability to afford health care.

c Availability score: weighted factor score of measures indicating the availability of health-care resources such as hospitals and specialists in the patient’s area.

d Accessibility score: weighted factor score of measures indicating the distance patient likely has to travel to care.

e Mortality risk is modeled starting from 1-year post diagnosis onward.

f Includes both the surgery and chemotherapy variables.

### HCA and OC-specific mortality in the full cohort

In cause-specific Cox proportional hazards regression models adjusted for patient clinical and demographic factors (including tumor stage and histology), patients with higher affordability (HR = 0.90, 95% CI = 0.87 to 0.94), availability (HR = 0.95, 95% CI = 0.92 to 0.99), and accessibility scores (HR = 0.93, 95% CI = 0.87 to 0.99) had a lower risk of death from OC compared with patients with lower scores (Table 3). After adjustment for all 3 HCA dimension scores, patient demographics, and clinical characteristics, only higher affordability scores retained a statistically significant association with reduced risk of OC mortality (HR = 0.91, 95% CI = 0.87 to 0.95) (Table 3). In fully adjusted models, NH Black patients had a 26% higher risk of OC mortality than NH White patients (HR = 1.26, 95% CI = 1.11 to 1.43). As with all-cause mortality, the NH Black vs NH White disparity was larger for OC when the cohort was limited to patients diagnosed with stage IV disease (HR = 1.36, 95% CI = 1.15 to 1.61) (Table 3). Hispanic patients had similar rates of OC-specific mortality as NH White patients in the full cohort and among patients with advanced-stage disease. No evidence of statistical interaction between patient race and ethnicity and HCA dimensions were observed.

**Table 3.** Association of race and ethnicity and HCA dimensions with risk of ovarian cancer mortality (HR and 95% confidence intervals) in the full cohort (N = 7590), advanced stage disease (n = 2812), and treatment receipt subcohort surviving at least 1 year (n = 4629)

	Patient characteristics^a^	Patient characteristics + affordability score^b^	Patient characteristics + availability score^c^	Patient characteristics + accessibility score^d^	Patient characteristics + 3 HCA scores	Estimated percentage of Non-Hispanic Black/Non-Hispanic White survival disparity explained
Full cohort						
Affordability score		0.90 (0.87 to 0.94)			0.91 (0.87 to 0.95)	26.0%
Availability score			0.95 (0.92 to 0.99)		0.97 (0.93 to 1.02)	0%
Accessibility score				0.93 (0.87 to 0.99)	0.97 (0.91 to 1.04)	−2.0%
Race (ref Non-Hispanic White)						
Hispanic	1.11 (0.96 to 1.27)	1.04 (0.91 to 1.19)	1.10 (0.96 to 1.26)	1.11 (0.97 to 1.27)	1.04 (0.91 to 1.20)	
Non-Hispanic Black	1.34 (1.18 to 1.51)	1.25 (1.10 to 1.41)	1.34 (1.18 to 1.51)	1.35 (1.20 to 1.53)	1.26 (1.11 to 1.43)	
Advanced stage at diagnosis (stage IV)						
Affordability score		0.90 (0.85 to 0.95)			0.90 (0.85 to 0.96)	18.2%
Availability score			0.96 (0.90 to 1.02)		0.98 (0.92 to 1.05)	−2.0%
Accessibility score				0.99 (0.89 to 1.10)	0.99 (0.89 to 1.10)	−2.0%
Race (ref Non-Hispanic White)						
Hispanic	1.06 (0.87 to 1.28)	1.00 (0.83 to 1.22)	1.07 (0.88 to 1.29)	1.07 (0.88 to 1.29)	1.01 (0.83 to 1.22)	
Non-Hispanic Black	1.44 (1.22 to 1.70)	1.36 (1.15 to 1.60)	1.45 (1.23 to 1.72)	1.45 (1.23 to 1.72)	1.36 (1.15 to 1.61)	
Survived at least 1-year post diagnosis^e^						
Affordability score		0.96 (0.90 to 1.02)			0.98 (0.92 to 1.05)	11.1%
Availability score			0.94 (0.87 to 1.02)		0.94 (0.87 to 1.02)	0%
Accessibility score				0.90 (0.81 to 1.00)	0.91 (0.82 to 1.02)	−4.4%
Race (ref Non-Hispanic White)						
Hispanic	0.83 (0.65 to 1.07)	0.81 (0.63 to 1.05)	0.82 (0.64 to 1.06)	0.84 (0.66 to 1.08)	0.82 (0.64 to 1.06)	
Non-Hispanic Black	1.45 (1.17 to 1.80)	1.40 (1.13 to 1.75)	1.45 (1.17 to 1.8)	1.47 (1.18 to 1.82)	1.45 (1.16 to 1.81)	
Consulted gynecologic oncologist	0.92 (0.81 to 1.04)	0.92 (0.81 to 1.05)	0.92 (0.81 to 1.05)	0.92 (0.81 to 1.04)	0.92 (0.81 to 1.05)	
Distance in miles to surgical facility	1.00 (1.00 to 1.00)	1.00 (1.00 to 1.00)	1.00 (1.00 to 1.00)	1.00 (1.00 to 1.00)	1.00 (1.00 to 1.00)	
Surgery met guidelines (ref No)	0.89 (0.79 to 0.99)	0.89 (0.79 to 1.00)	0.89 (0.79 to 1)	0.89 (0.80 to 1.00)	0.89 (0.80 to 1.00)	18.0% (all treatment variables)^f^
Completed recommended cycles of chemotherapy (ref No)	0.94 (0.84 to 1.05)	0.94 (0.84 to 1.05)	0.94 (0.84 to 1.05)	0.94 (0.84 to 1.05)	0.94 (0.84 to 1.05)	

a Patient age at diagnosis, stage at diagnosis, tumor histology, region of residence, and comorbid conditions. HCA = health-care access; HR = hazard ratio.

b Affordability score: weighted factor score of measures indicating patient’s ability to afford health care.

c Availability score: weighted factor score of measures indicating the availability of health-care resources such as hospitals and specialists in the patient’s area.

d Accessibility score: weighted factor score of measures indicating the distance patient likely has to travel to care.

e Mortality risk is modeled starting from 1-year post diagnosis onward.

f Includes both the surgery and chemotherapy variables.

### HCA and all-cause and OC-specific mortality in the treatment receipt subcohort

**Figure 4.**
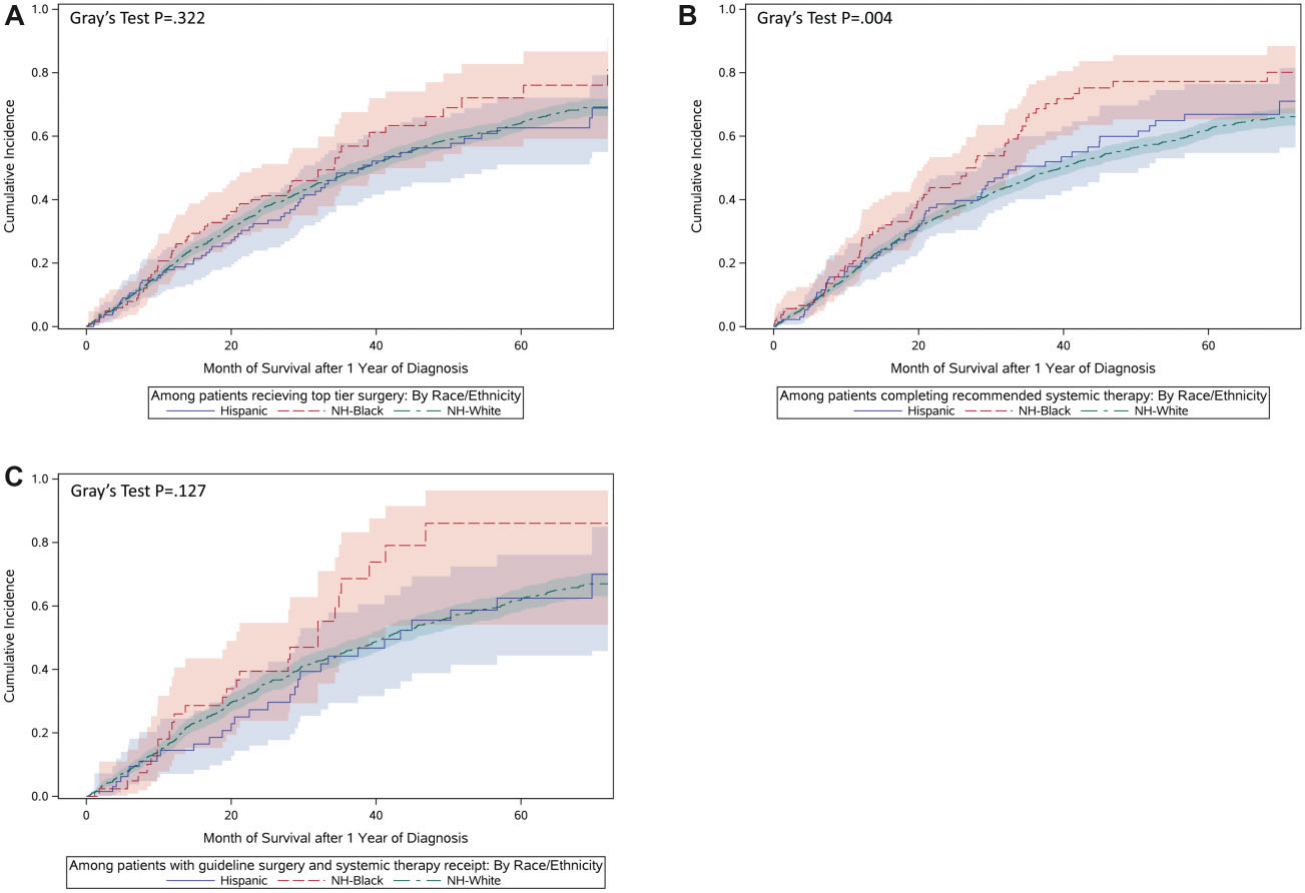
**A)** Cumulative incidence of mortality by race and ethnicity (Hispanic, non-Hispanic [NH] Black, NH White) among patients who received guideline-adherent surgery (N = 2292). **B)** Cumulative incidence of mortality by race and ethnicity (Hispanic, NH Black, NH White) among patients who completed guideline-recommended systemic therapy (N = 2265). **C)** Cumulative incidence of mortality by race and ethnicity (Hispanic, NH Black, NH White) among patients who received guideline-adherent surgery and completed guideline-recommended systemic therapy (N = 1199).

In the subcohort of patients who survived at least 12 months, receipt of guideline-concordant surgery was associated with decreased risk of all-cause mortality (HR = 0.90, 95% CI = 0.81 to 0.99) (Table 2); the addition of the 3 HCA dimension scores did not change the estimate (HR = 0.90, 95% CI = 0.82 to 1.00). A similar association was observed for OC-specific mortality (HR = 0.89, 95% CI = 0.79 to 0.99) and did not change after additional adjustment for the 3 HCA dimension scores (Table 3). Completion of the recommended cycles of systemic therapy was associated with lower all-cause mortality (HR = 0.89, 95% CI = 0.81 to 0.98), but not lower OC mortality. Figure 4 presents unadjusted cumulative incidence functions of OC mortality stratified by patient race and ethnicity among patients who received guideline treatment. In fully adjusted Cox proportional hazards models, NH Black patients had a 24% higher risk of all-cause mortality (HR = 1.24, 95% CI = 1.01 to 1.52) and a 45% higher risk of OC mortality compared with NH White patients (HR = 1.45, 95% CI = 1.16 to 1.81) after adjusting for guideline-concordant treatment, consultation with a gynecologic oncologist, distance to care, and all HCA dimensions, although none of the HCA dimensions remained statistically significant. No evidence of statistical interaction between patient race and ethnicity and HCA dimensions was observed.

In analysis of excess risk associated with HCA dimensions and treatment, affordability explained 32% (all-cause) and 26% (OC-specific) of the NH Black versus NH White survival disparity in the full cohort after accounting for patient characteristics (Tables 2and 3). Guideline-concordant treatment receipt explained 7% of the all-cause mortality disparity and 18% of the OC-specific mortality disparity in the treatment receipt subcohort (Tables 2and 3).

## Discussion

In this retrospective cohort study of patients aged at least 65 years with OC identified in SEER-Medicare, higher HCA dimension scores were statistically significantly associated with decreased all-cause and OC-specific mortality. Higher affordability (eg, living in a higher SES census tract) remained statistically significantly associated with decreased mortality after adjustment for demographic and clinical factors and the other HCA dimensions and accounted for the greatest proportion of the observed disparity. Notably, disparities in survival among NH Black patients persisted even after accounting for HCA and demographic and clinical factors. In the subcohort of patients who survived at least 1 year after diagnosis (treatment receipt subcohort), the racial disparity was larger, even after accounting for HCA dimensions, guideline-concordant treatment, consultation with a gynecologic oncologist, and distance to care, with a 45% higher risk of OC death in NH Black patients. These data address an important gap in the scientific literature by estimating the impact of multiple dimensions of HCA on survival in a diverse OC patient population while comprehensively accounting for demographic and clinical factors. Our results indicate that efforts to address gaps in HCA to enhance equity in OC treatment are critical, particularly for NH Black patients, even if these factors do not fully explain observed racial disparities. Additional research on other HCA dimensions and potential biological mechanisms driving these disparities are urgently needed.

In line with prior work, our study affirms that disparities exist with respect to HCA for patients with OC (14,22): NH Black and Hispanic patients had lower HCA scores for affordability and availability. Though distance to treatment (ie, accessibility) may be shorter for NH Black and Hispanic patients, other accessibility-related barriers to care not assessed in this study (eg, reliable transportation) may be present. Certain accessibility and availability metrics specific to gynecologic cancers were additionally considered in relation to survival, including consultation with a gynecologic oncologist, which has been associated with an increased likelihood of surgery receipt (23) and improved survival (24). Though we could not assess distance to a gynecologic oncologist—which has been associated with survival (25)—we did assess distance to treatment facility. After accounting for receipt of guideline-adherent treatment, neither consultation with a gynecologic oncologist nor distance to treatment facility were associated with survival. Overall, higher affordability, availability, and accessibility scores were associated with decreased risk of mortality in the full cohort, highlighting that strategies to equalize access based on these dimensions can substantially improve OC outcomes for all patients. Affordability is particularly important, because it explained 26%-32% of the survival disparities between NH Black and NH White patients. Interestingly, Hispanic patients demonstrated similar HCA dimension scores to NH Black patients but had a mortality risk profile more similar to the NH White patients. Additional studies are needed to explore this well-described “Hispanic-paradox” phenomenon, where Hispanics in the United States experience greater disadvantage but have similar or better health outcomes compared with NH Whites (26).

Prior work has demonstrated mixed results regarding the impact of treatment utilization on racial and ethnic disparities: some studies have shown that receipt of guideline-adherent treatment leads to equivalent survival across racial groups (27-29), whereas other studies have demonstrated that disparities persist even with receipt of guideline-adherent treatment (4,30). In our study, NH Black patients were less likely to receive guideline-adherent surgery than NH White and Hispanic patients. No differences were observed in rates of initiating systemic therapy, though NH Black patients were less likely to complete their treatment. Ultimately, the mortality disparities among NH Black patients persisted after adjustment for receipt of guideline-based treatment among a subcohort of patients who were alive for at least 12 months post diagnosis to allow for sufficient time to initiate or complete therapy. In that cohort, we observed that receipt of guideline-recommended surgery and chemotherapy accounted for 7%-18% of the survival disparities between NH Black and NH White patients. We suspect that low rates of guideline-concordant treatment among NH Black patients may be partly driven by the influence of other patient-level factors such as comorbidities, older age, and frailty in patients with advanced-stage disease—measures that are difficult to accurately capture in claims datasets (31). Additionally, comorbidities and more aggressive tumor phenotypes may contribute to the higher risk of mortality observed for NH Black patients, even after accounting for guideline-concordant care. In our study, NH Black patients had a higher comorbidity burden and were more likely to have hypertension, congestive heart failure, diabetes, peripheral vascular disease, end-stage renal disease, and chronic obstructive pulmonary disease than NH White patients. Although medical comorbidities and frailty may contribute to lower rates of guideline-concordant treatment, providing additional support to those with comorbidities and frailty in the form of palliative care services, coordination of care, and geriatric assessments may contribute to more tolerable treatment and improve adherence to care for these individuals. Additionally, other studies have shown that Black patients are more likely to have platinum-resistant disease and are less likely to have optimal tumor cytoreductive surgeries than White patients (32), highlighting the need for future studies to characterize OC molecular biomarkers among racially diverse populations.

Two HCA dimensions remain unaccounted for in our study as they cannot be estimated in the SEER-Medicare dataset: accommodation (coordination of care) and acceptability (quality of patient–provider interaction) (12). These are measures of patient-centered care, including communication, cultural competence, implicit bias, and discrimination, which may be important moderators of the association between other HCA dimensions and treatment outcomes, especially for NH Black patients. For example, perceived discrimination is associated with decreased health-care use and adherence to medical recommendations (33,34) and prolonged symptom duration before OC diagnosis among NH Black women (35). Conversely, the use of patient-reported outcome measures has been associated with higher patient acceptability and improved patient satisfaction, health outcomes, and perception of care quality (36-38). Although validated measures of organizational accommodation exist, these should be refined to assess cancer care specifically (39). In addition, few studies that examine racial disparities in gynecologic cancers integrate measures of structural racism, such as residential segregation and housing discrimination (40,41). Future studies should incorporate measures of structural racism in concert with patient-level surveys on organizational accommodation and patient-reported outcome measures (including discrimination) to better characterize health-care accommodation and acceptability in relation to treatment receipt and outcomes. Disparities that persist after adjusting for these measures suggest the contribution of molecular factors—potentially a result of ancestry and exposure to social factors—which also deserve further study.

There are several limitations to this study. With respect to our analysis of HCA, many of the variables comprising the HCA dimension scores are calculated at the level of a given census tract, referral region, or county. More granular individual patient-level data are not available, and these metrics were used as the best available proxy to gain insight into a patient’s HCA status. However, these regional metrics may differ substantially from that of an individual patient’s true ability to access quality health care. Additionally, these data capture receipt of surgery and initiation or completion of recommended therapies with reasonable reliability. However, there are likely other factors influencing care quality for which the available data are unable to account. Our analysis relies on Medicare claims datasets, limiting our analysis to patients aged 65 years and older; our requirement for continuous enrollment for over 1 year further restricted our age cohort to 66 years and older, which limits the generalizability of our study to younger OC patients, though nearly one-half of patients are diagnosed at ages older than 63 years (1). Additionally, our treatment receipt subcohort was limited to patients surviving 1 year. This design was intended to allow for analysis of claims data but also to negate any impact of late diagnosis and/or extremely aggressive histology that may have been less amenable to guideline-adherent treatment. However, this inherently compromises our ability to make meaningful inferences about disparities that likely exist in the receipt of treatment within the first year after diagnosis as well as disparities in the number of patients surviving 1 year. Additionally, though the excess risk attributable to HCA dimensions and treatment receipt in relation to the NH Black/NH White survival disparity offers clinical perspective, the measure does not account for the precision of the estimates and should be interpreted with caution.

Higher HCA affordability, availability, and accessibility were statistically significantly associated with decreased risk of mortality after OC, though racial disparities persisted even after accounting for these measures. Although equalizing access to health care remains critical, the dimensions evaluated in this study do not fully capture the etiology of worse outcomes for NH Black patients with OC. Further work on other HCA dimensions and tumor biology is needed to determine other factors contributing to OC survival disparities.

## Supplementary Material

pkad011_Supplementary_Data

## Data Availability

The SEER-Medicare database is owned and managed by the National Cancer Institute. Information on how to obtain these data is available at: https://healthcaredelivery.cancer.gov/seermedicare/obtain/.
