# Supplementary material for: Health-care access dimensions and ovarian cancer survival: SEER-Medicare analysis of the ORCHiD study
Source: JNCI Cancer Spectr. 2023 Feb 16;7(2):pkad011. doi: 10.1093/jncics/pkad011 (PMC10066801; doi:10.1093/jncics/pkad011)
Supplement: pkad011_Supplementary_Data [file pkad011_supplementary_data.docx]

**SUPPLEMENTARY MATERIAL**

**Supplementary Figure 1: Decision Tree for Guideline-Concordant Primary Treatment for Epithelial Ovarian Cancer**

All patients (age 65+ years at diagnosis)

Borderline Epithelial histology

Type I/II Epithelial histology or carcinosarcoma

Comprehensive staging laparotomy, including total abdominal hysterectomy (if no prior hysterectomy), bilateral salpingo-oophorectomy and lymph node dissection + omentectomy + pelvic or para-aortic lymph node biopsy (for Stage I-IIIB)

Observation (chemotherapy not required)

Excluded from all analyses of treatment receipt

Stage IA/IB

Stage IC/II-IV

**Stage IC:**

IV platinum doublet for at least 3 cycles*

**Stage II/III**:

Intraperitoneal chemotherapy or hyperthermic intraperitoneal chemotherapy (HIPEC) or at least 6 cycles† of IV platinum doublet

**Stage IIIC/IV before or after surgery:**

At least 6 total cycles† of IV platinum doublet

**Grade 1:**

Observe (excluded from chemotherapy analysis)

**Grade 2:**

Observe or IV platinum doublet for at least 3 cycles* (exclude from chemotherapy analysis)

**Grade 3 or clear cell histology:**

IV platinum doublet for at least 3 cycles*

Simplified and adapted from NCCN Ovarian Cancer Clinical Guidelines version 2.2013; this diagram does not reflect the full NCCN recommendations and is limited to clinical information and treatments that can be assessed in the SEER-Medicare database

*NCCN guidelines specifically recommend 3-6 cycles of IV platinum doublet

†NCCN guidelines specifically recommend 6-8 cycles of IV platinum doublet

**Supplementary Figure 2: Decision Tree for Guideline-Concordant Primary Treatment for Less Common Histologic Types**

All patients (age 65+ years at diagnosis)

Comprehensive staging laparotomy, including total abdominal hysterectomy and bilateral salpingo-oophorectomy + omentectomy + (for Stage I-IIIB germ cell pelvic or para-aortic lymph node biopsy)

Simplified and adapted from NCCN Ovarian Cancer Clinical Guidelines version 2.2013; this diagram does not reflect the full NCCN treatment recommendations and is limited to initial treatment recommendations and clinical information/treatments that can be assessed in the SEER-Medicare database

*NCCN guidelines specify initial observation for Stage I dysgerminoma, but also specify that 3 courses of etoposide/carboplatin is appropriate for certain patients with Stage IB-III disease. In this study, we do not require Stage IB/IC dysgerminoma patients to receive chemotherapy to be guideline concordant as we cannot identify the patient population for which it is appropriate in this database.

†NCCN guidelines specifically recommend 6-8 cycles of IV platinum doublet

3-4 cycles with bleomycin/ etoposide/ platinum (BEP)

Or at least 6 cycles††of paclitaxel/ carboplatin

Observe

(exclude from chemotherapy analysis)

Stage II-IV

Stage I

3-4 cycles with bleomycin/

etoposide/

platinum (BEP) or platinum doublet

Or 3 cycles* of etoposide/ carboplatin (Stage II-III only)

Stage II-IV Dysgerminoma

3-4 cycles with bleomycin/ etoposide/

platinum (BEP)

Observe

(exclude from chemotherapy analysis)

Stage I, grade 2 or Stage II-IV immature teratoma,

Or any stage embryonal or endodermal

Stage 1 Dysgerminoma or Stage 1 grade 1 immature teratoma

Malignant Sex Cord Stromal histology

Malignant Germ Cell histology

| **Supplementary Table 1. Baseline patient measures of healthcare Affordability, Accessibility, and Availability at time of ovarian cancer diagnosis by race and ethnicity in full ORCHiD cohort (N=8,987)** | | | | |
| --- | --- | --- | --- | --- |
|  | **Non-Hispanic White** | **Non-Hispanic Black** | **Hispanic** | **p-value** |
| **Total** | **7,822** | **612** | **553** |  |
| **Categorical Variables. N (%)** |  |  |  |  |
| **AFFORDABILITY MEASURES** |  |  |  |  |
| Patient is dual enrolled in Medicaid and Medicare | 870 (11.2) | 255 (41.7) | 273 (49.4) | <0.001 |
| Patient's primary hospital eligibility for disproportionate share payments | 5853 (74.8) | 518 (84.6) | 481 (87.0) | <0.001 |
| **ACCESSIBILITY MEASURES** |  |  |  |  |
| Patient lives in metropolitan area | 6535 (83.6) | >540 | >500 | <0.001 |
| Patient lives in a metropolitan or metropolitan-adjacent area | 7280 (93.1) | 600 (98.0) | 535 (96.8) | <0.001 |
| Patient lives in rural area | 174 (2.2) | <11 | <11 | 0.001 |
| Patient's main hospital is rural primary hospital | 364 (4.7) | 13 (2.1) | <11 | <0.001 |
| **AVAILABILITY MEASURES** |  |  |  |  |
| Patient's main hospital teaching status | 3941 (50.4) | 400 (65.4) | 295 (53.4) | <0.001 |
| Patient's main hospital NCI cancer center designation |  |  |  | 0.004 |
| Clinical | 178 (2.3) | <11 | 25 (4.5) |  |
| Comprehensive | 507 (6.5) | <50 | 33 (6.0) |  |
| Patient's main hospital is member of NCI gynecologic oncology group | 1629 (20.8) | 139 (22.7) | 108 (19.5) | 0.393 |
| Specialty of patient’s primary cancer physician |  |  |  | <0.001 |
| General surgery | 97 (1.2) | <11 | <11 |  |
| Gynecologic oncology | 1803 (23.1) | 146 (23.9) | 112 (20.3) |  |
| Hematology/oncology/medical oncology | 3629 (46.4) | 220 (36.0) | 258 (46.7) |  |
| Internal medicine | 793 (10.1) | 86 (14.1) | 73 (13.2) |  |
| No primary | 184 (2.4) | 29 (4.7) | 20 (3.6) |  |
| Ob-Gyn | 446 (5.7) | 49 (8.0) | 30 (5.4) |  |
| Other | 324 (4.1) | 42 (6.9) | 20 (3.6) |  |
| Pathology/other oncology | 99 (1.3) | <11 | <20 |  |
| Primary/general | 430 (5.5) | 24 (3.9) | 23 (4.2) |  |
| Surgical oncology | 17 (0.2) | 0 | 0 |  |
| **Continuous Variables, Mean (SD)** |  |  |  |  |
| **AFFORDABILITY MEASURES** |  |  |  |  |
| Census tract at diagnosis: Percent Black residents | 7.6 (13.3) | 50.6 (33.2) | 6.1 (9.9) | <0.001 |
| Census tract at diagnosis: Percent persons 25+ with at least 4 years of college | 33.0 (18.9) | 20.4 (14.8) | 24.4 (17.6) | <0.001 |
| Census tract at diagnosis: Median household income | 67757.9 (31658.8) | 45296.9 (23609) | 57021.6 (26982.3) | <0.001 |
| Census tract at diagnosis: Percent persons 25+ with <high school education | 11.6 (9.1) | 19.5 (10.7) | 22.7 (16.4) | <0.001 |
| Census tract at diagnosis: Per capita income for Census tract | 34,327.4 (17108.3) | 22,026.4 (9964.6) | 26577.1 (14668.5) | <0.001 |
| Census tract at diagnosis: Percent persons 25+ with some college education | 29.3 (7.9) | 28.6 (7.8) | 28.4 (8.7) | 0.007 |
| Census tract at diagnosis: Percent of households below poverty line | 11.8 (9.2) | 23.0 (13.3) | 17.5 (11.7) | <0.001 |
| County level: Percent of residents without health insurance | 14.0 (5.0) | 15.9 (4.6) | 16.2 (5.1) | <0.001 |
| **ACCESSIBILITY MEASURES** |  |  |  |  |
| Straight line geographic distance from patient residential zip code to patient's main hospital zip code | 21.2 (119.0) | 21.5 (150.2) | 20.1 (125.4) | 0.979 |
| County level: # hospitals per 1K residents in patient’s county in year of diagnosis | 2.0 (2.1) | 2.2 (2.1) | 1.5 (1.6) | <0.001 |
| **AVAILABILITY MEASURES** |  |  |  |  |
| Patient's main hospital number of beds | 429.0 (4467.0) | 395.2 (253.3) | 332.6 (202.2) | 0.861 |
| HRR level: Discharges for ambulatory sensitive conditions per 1K population | 57.5 (18.4) | 65.7 (16.5) | 51.4 (13.7) | <0.001 |
| HRR level: Hematologists/oncologists per 100K residents | 3.3 (0.9) | 3.3 (0.9) | 3.1 (0.8) | <0.001 |
| HRR level: Percentage of Medicare beneficiaries that died in year of diagnosis | 4.4 (0.6) | 4.6 (0.6) | 4.0 (0.5) | <0.001 |
| HRR level: Hospital-based physicians per 100K residents (2011) | 25.6 (2.8) | 24.4 (2.6) | 25.6 (2.4) | <0.001 |
| HRR level: Ob-Gyn per 100K women | 60.2 (14.9) | 60.3 (12.0) | 56.5 (14.8) | <0.001 |
| HRR level: Percentage of Medicare beneficiaries seeing a PCP that year | 77.1 (4.8) | 77.2 (5.0) | 73.9 (4.6) | <0.001 |
| HRR level: PCPs per residents | 74.7 (11.2) | 72.1 (9.6) | 73.9 (12.1) | <0.001 |
| HRR level: Hospital discharge 30 day return to ER rates (%) | 19.6 (1.4) | 19.7 (1.3) | 19.3 (1.5) | <0.001 |
| HRR-level: 30 days hospital readmission rates (%) | 15.5 (1.2) | 16.1 (1.0) | 15.4 (1.2) | <0.001 |
| HRR level: Physicians per 100K residents | 210.6 (30.5) | 206.8 (28.5) | 206.4 (32.0) | <0.001 |
| HRR level: Surgeons per 100K residents | 42.1 (6.4) | 41.6 (5.4) | 40.1 (7.0) | <0.001 |
| County level: # Gynecologic-oncologists per 1K residents in year of diagnosis | 12.1 (7.4) | 15.4 (8.4) | 11.9 (5.9) | <0.001 |
| County level: # Ob-Gyns seeing patients per 1K residents | 11.8 (7.2) | 14.9 (8.2) | 11.6 (5.7) | <0.001 |
| County level: # PCPs per 1K residents | 76.0 (28.0) | 77.2 (26.4) | 72.6 (24.0) | 0.011 |
| Abbreviations: standard deviation (SD); obstetrician-gynecologist (Ob-Gyn); primary care physician (PCP); National Cancer Institute (NCI); emergency room (ER); ovarian cancer (OC); healthcare referral region (HRR). | | | | |

| **Supplementary Table 2. Factor loadings and reliability test from the two stage 3 factor solution for original and adjusted factors** | | | | | | | |  |
| --- | --- | --- | --- | --- | --- | --- | --- | --- |
|  |  | **Original** | **Latent factor’s composite reliability (Ω) (>0.7)** | **Latent factor’s average variance extracted (AVE) (>0.5)** | **Adjusted** | | **Latent factor’s composite reliability (Ω) (>0.7)** | **Latent factor’s average variance extracted (AVE) (>0.5)** |
| **Factor 1** |  |  |  |  |  | |  |  |
| HRR: Hematologists/oncologists per 100,000 residents (2011) | | 0.692 | 0.848 | 0.636 | | 0.692 | 0.848 | 0.637 |
| HRR: Hospital-based physicians per 100,000 residents (2011) | | 0.577 |  |  | | 0.58 |  |  |
| HRR: Primary care physicians per 100,000 residents (2011) | | 0.806 |  |  | | 0.806 |  |  |
| HRR: Total physicians per 100,000 residents (2011) | | 1 |  |  | | 1 |  |  |
| HRR: Surgeons per 100,000 residents (2011) |  | 0.735 |  |  | 0.735 | |  |  |
| **Factor 2** |  |  |  |  |  | |  |  |
| Census tract at diagnosis: Percent persons 25+ with at least 4 years of college |  | 0.896 | 0.876 | 0.689 | 0.896 | | 0.876 | 0.689 |
| Census tract at diagnosis: Median household income | | 0.897 |  |  | | 0.896 |  |  |
| Census tract at diagnosis: Percent persons 25+ with <12 year education | | 0.714 |  |  | | 0.714 |  |  |
| Census tract at diagnosis: Per capita income for Census tract | | 0.926 |  |  | | 0.926 |  |  |
| Census tract at diagnosis: % of households below poverty line | | 0.683 |  |  | | 0.683 |  |  |
| **Factor 3** |  |  |  |  |  | |  |  |
| Patient residence in a metropolitan or metropolitan-adjacent area |  | 0.664 | 0.394 | 0.396 | 0.649 | | 0.798 | 0.634 |
| Patient lives in metropolitan area |  | 0.861 |  |  | 0.937 | |  |  |
| Patient's main hospital is rural primary | | 0.464 |  |  | | 0.401 |  |  |
| County level: # hospitals per 1K residents in year of diagnosis | | -0.629 |  |  | | - |  |  |

**Supplementary Table 3: Coding definitions for guideline-concordant surgery**

| **High confidence that surgery met guideline recommendations** | |
| --- | --- |
| **CPT** |  |
| 58943 | Oophorectomy, partial or total, unilateral or bilateral; for ovarian malignancy, with para-aortic and pelvic lymph node biopsies, peritoneal washings, peritoneal biopsies, diaphragmatic assessment, with or without salpingectomy(s), with or without omentectomy |
| 58950 | Resection of ovarian malignancy with bilateral salpingo-oophorectomy and omentectomy |
| 58951 | Resection of ovarian malignancy with bilateral salpingo-oophorectomy and omentectomy, with abdominal hysterectomy, pelvic and limited para-aortic lymphadenectomy) |
| 58952 | Resection of ovarian malignancy with bilateral salpingo-oophorectomy and omentectomy, with radical dissection for debulking |
| 58953 | Bilateral salpingo-oophorectomy with omentectomy, total abdominal hysterectomy and radical dissection for debulking |
| 58954 | Bilateral salpingo-oophorectomy with omentectomy, total abdominal hysterectomy and radical dissection for debulking, with pelvic lymphadenectomy and limited para-aortic lymphadenectomy) |
| 58960 | Laparotomy for staging or restaging of ovarian, tubal or primary peritoneal malignancy (second look) with or without omentectomy, peritoneal washing, biopsy of abdominal and pelvic peritoneum, diaphragmatic assessment with pelvic and limited per-aortic lymphadenectomy |
| \| **SEER primary site surgical codes** \| \| \| --- \| --- \| \|  \| 55, 56, 57, 70, 71, 72, 73, 74 \| |  |

**Supplementary Table 4: Codes for any systemic therapy and recommended systemic therapies**

| **Any Systemic Therapy** | **CPT:** 964xx, 965xx, Q0083-Q0085, G0355-G0362, J8530-J8999 and J9xxx |
| --- | --- |
|  | **ICD-9 and ICD-10 Procedure codes:**  V58.1, V66.2, V62.7, E9331, E9307, 99.25  Z5111, Z5112, Z5189, 3E03305, 3E04305, XW03351, XW033B3, XW033C3, XW04351, XW043B3, XW043C3 |
| **Intraperitoneal chemotherapy** | **CPT:** 96445 |
| **Platinum Doublets** |  |
| **Carboplatin or Cisplatin** | **CPT:** J9045, J9062, J9060, C9418 |
|  | **NDC**: 477810605, 477810606, 477810604, 000153210, 000153211, 000153212, 000153213, 000153214, 000153215, 000153216, 551500386, 167290295, 167290295, 167290295, 167290295, 477810603, 007034239, 007034239, 007034244, 007034244, 007034246, 007034246, 007034248, 007034248, 007034248, 572770105, 572770106, 572770107, 633230172, 633230172, 633230172, 633230172, 674570491, 674570492, 674570493, 674570494, 674570608, 694480005, 694480005, 694480005, 694480005, 694480005, 007033249, 250210202, 250210202, 250210202, 250210202, 473350150, 473350151, 473350284, 507420445, 507420446, 507420447, 507420448, 617030339, 617030339, 617030339, 617030339, 667580047, 667580047, 667580047, 667580047, 680830190, 680830191, 680830192, 680830193, 712880100, 712880100, 712880100, 712880100, 617030360, 617030360, 617030360, 674570424, 674570425, 708600206, 708600206, 000690081, 000690084, 007035747, 007035748, 167290288, 167290288, 445670509, 445670510, 445670511, 477810609, 477810610, 611260003, 611260004, 611260004, 633230103, 633230103, 633230103, 680010283, 680010283, 680830162, 680830163, 445670530, 000153070, 000153072, 000153072 |
| **Second agents for platinum doublets** | **CPT:** C9127, C9431, J9264, J9265, J9267, J9170, J9171, J9091, J9070, J9092, J9080, J9090, C9420, C9421, J9093, J9094, J9095, J9096, J9097, J8530, J9201, J9350, J8705, J9351, J9000, C9415, J9002, Q2048, Q2049, Q2050, J9001 |
|  | **NDC:** 477810593, 477810594, 477810595, 459630613, 459630613, 459630613, 459630613, 459630613, 459630613, 007034764, 007034764, 007033216, 007033216, 007033217, 007033213, 007033213, 007033218, 007033218, 167140137, 695390158, 695390159, 695390157, 708600215, 722050063, 722050062, 722050061, 000690076, 000690078, 000690079, 007034766, 007034766, 007034767, 007034768, 007034768, 250210213, 250210213, 250210213, 445670504, 445670505, 445670506, 519910937, 519910938, 553900114, 553900304, 553900314, 617030342, 617030342, 617030342, 633230763, 633230763, 633230763, 633230763, 633230763, 633230763, 667580043, 667580043, 667580043, 674570434, 674570449, 674570471, 680830178, 680830179, 680830180, 688170134, 708600200, 708600200, 708600200, 701211221, 701211222, 701211223, 430660001, 430660006, 430660010, 435980389, 473350323, 473350895, 473350939, 724850216, 724850215, 724850214, 712880143, 712880144, 712880144, 712880150, 712880151, 712880151, 000699144, 000699144, 004090369, 674570531, 674570532, 674570781, 690970369, 690970371, 001439204, 001439205, 000699141, 000699141, 000699142, 000699142, 000758001, 000758001, 004090201, 004090201, 004090201, 004090201, 004090201, 004090201, 004097870, 004090365, 004091732, 004094235, 004095068, 551500378, 551500379, 551500380, 680830401, 680830400, 680830399, 707000176, 707000175, 707000174, 000758003, 000758004, 000758005, 004090366, 004090367, 004090368, 007035720, 007035730, 009551020, 009551021, 009551022, 167140465, 167140500, 167290120, 167290228, 167290231, 167290231, 167290231, 167290267, 167290267, 167290267, 250210222, 250210222, 250210222, 398222120, 398222180, 398222200, 423670121, 423670121, 423670121, 435980258, 435980259, 435980610, 435980611, 459630734, 459630734, 459630734, 459630765, 459630781, 459630790, 578843021, 637390932, 637390971, 667580050, 667580050, 667580050, 667580950, 667580950, 667580950, 250210245, 250210245, 473350285, 507420428, 507420431, 507420463, 701211240, 701211239, 701211238, 548790022, 548790021, 439750308, 439750307, 000150502, 167140857, 167140858, 167140859, 507420519, 507420520, 625590930, 625590931, 680010442, 680010443, 680010444, 726030104, 726030411, 726030326, 680010370, 680010371, 680010372, 690970516, 690970517, 100190982, 100190984, 708600218, 708600218, 000540382, 000540383, 000544129, 000544130, 007813233, 007813244, 007813255, 100190935, 100190936, 100190937, 100190938, 100190939, 100190942, 100190943, 100190944, 100190945, 100190955, 100190956, 100190957, 100190988, 100190989, 100190990, 548685005, 548685218, 548685218, 691890382, 691890383, 000150505, 000150503, 000150504, 000150506, 167290391, 167290419, 167290423, 605056113, 605056114, 605056115, 674570616, 674570617, 674570618, 680010359, 680010350, 680010348, 680010342, 167140909, 167140930, 250210239, 250210239, 250210239, 507420496, 507420497, 507420498, 633230102, 633230102, 637593028, 637593029, 627560008, 627560073, 627560102, 627560219, 627560321, 627560438, 627560533, 627560614, 627560746, 627560974, 712880113, 712880114, 459630623, 459630624, 459630636, 167290426, 724850221, 724850222, 724850223, 000027501, 004090181, 004090181, 004090182, 004090182, 004090183, 004090183, 004090185, 004090187, 250210209, 250210234, 250210235, 422360001, 422360002, 459630612, 459630619, 459630620, 551110686, 551110687, 633230125, 633230125, 633230125, 633230126, 633230126, 708600204, 708600205, 712880117, 712880117, 712880117, 007035775, 007035778, 000027502, 000693857, 000693858, 000693859, 004090186, 005913562, 005913563, 007813282, 007813283, 167290092, 167290117, 167290118, 231550213, 231550214, 231550483, 231550484, 231550528, 231550529, 250210208, 473350153, 473350154, 553900391, 553900391, 674570462, 674570463, 674570464, 680010282, 680010282, 680010282, 680010282, 680830148, 680830149, 690970313, 690970314, 001439394, 001439395, 674570662, 167290243, 167290243, 000780672, 000780673, 004090302, 004090302, 507420404, 007034714, 007034714, 250210236, 667580051, 667580051, 667580051, 000780674, 167290151, 250210206, 250210206, 250210824, 459630615, 553900370, 627560023, 627560023, 633230762, 633230762, 633230762, 633230762, 633230762, 664350410, 664350410, 674570474, 000074205, 000074207, 000690075, 000074201, 001439275, 001439277, 435980682, 435980683, 477810256, 477810256, 477810256, 680010345, 680010345, 553900237, 553900238, 167140001, 493150008, 493150009, 001439092, 001439093, 726030103, 726030200, 000690170, 000690171, 000693030, 000693034, 674570394, 674570394, 701211218, 701211218, 003380067, 003380063, 003380080, 003380086, 707101530, 707101531, 000131116, 000131136, 000131146, 000131156, 000131176, 000131266, 000131286, 000153352, 000153353, 680010492, 680010493, 000693031, 000693032, 000693033, 000694004, 000694015, 000694026, 000694030, 000694031, 000694032, 000694033, 000694034, 000694037, 001439546, 001439547, 001439548, 001439548, 001439549, 001439549, 004090124, 007035040, 007035043, 007035043, 007035046, 167140742, 167140856, 250210207, 250210207, 250210207, 435980283, 435980541, 459630733, 459630733, 459630733, 459630733, 473350049, 473350050, 473350082, 473350083, 531500314, 531500314, 531500315, 531500317, 531500320, 531500320, 596760960, 596760960, 596760966, 596760966, 627560826, 627560827, 633230101, 633230883, 633230883, 633230883, 674570393, 674570393, 674570395, 674570396, 674570436, 674570478, 680830248, 680830249, 680830250, 701211219 |
| **Etoposide** | **CPT:** J9181, J9182, C9425, J8560, C9414 |
|  | **NDC:** 007035657, 007035657, 633230104, 633230104, 633230104, 680010265, 680010265, 680010265, 680010265, 680010265, 680010265, 000153404, 003783266, 007035653, 007035656, 007035656, 167290114, 167290114, 167290114, 167290114, 167290262, 167290262, 553900291, 553900292, 553900293, 553900491, 553900492, 553900493 |
| **Bleomycin** | **CPT:** C9417, J9040 |
|  | **NDC:** 674570424, 674570425, 708600206, 708600206, 000690081, 000690084, 007035747, 007035748, 167290288, 167290288, 445670509, 445670510, 445670511, 477810609, 477810610, 611260003, 611260004, 611260004, 633230103, 633230103, 633230103, 680010283, 680010283, 680830162, 680830163, 445670530, 000153070, 000153072, 000153072 |

**Supplementary Methods: HCA measure definitions and creation of HCA dimension scores**

*Assignment of primary provider and hospital treatment facility:* A patient’s primary cancer treatment provider was identified as the provider listed on the highest number of the patient’s outpatient, carrier, home health, and hospice claims listing a cancer diagnosis. Physician specialties were determined from Medicare claims files using Health Care Financing Administration (HCFA) specialty codes. Ties between physicians were broken by prioritizing physician specialties of interest (gynecologic oncology, medical oncology, hematology/oncology, or surgical oncology) and claim date closest to the ovarian cancer (OC) diagnosis date. The patient’s primary treating hospital in the year the patient was diagnosed was defined as the facility at which the patient had the majority of inpatient and outpatient claims in that calendar year. In the case of ties, priority was given to facilities with records in the SEER-Medicare Hospital File.

*Measures of healthcare affordability:* Measures of healthcare affordability included dual enrollment in Medicaid, census tract-level measures of socioeconomic status (SES), and county-level health insurance coverage. A patient’s dual Medicaid enrollment status in the 12 months prior to OC diagnosis was sourced from the SEER-Medicare dataset, as were the following SES indicators of the patient’s residential census tract at the time of diagnosis drawn from data from the US Census Bureau’s American Community Survey: median per capita income, percentage of Black residents, percentage of adults 25+ with less than a high school education, percentage of households with incomes below the poverty level, and percentage of adults 25+ with a college degree. Census tract SES characteristics were categorized into quartiles, and included as a binary variable in models (highest quartile versus lower three quartiles). Federal Information Processing Standards (FIPS) codes for the patient’s county and state of residence and the patient’s year of diagnosis were used to link to the US Census Bureau’s Small Area Health Insurance Estimates 2008-2018 American Community Survey-Based Estimates datasets (https://www.census.gov/data/datasets/time-series/demo/sahie/estimates-acs.html) to obtain the estimated percentage of county residents without health insurance in the year of the patient’s diagnosis.

*Measures of healthcare availability:* Healthcare availability metrics for the patient’s county and healthcare referral regions were linked to SEER-Medicare data using year of diagnosis, county and state FIPS codes, and patient zip codes from the Area Healthcare Resources File and the Dartmouth Atlas Project. County-level metrics were drawn from the publicly available Area Healthcare Resource Files provided by the Health Resources and Services Administration (<https://data.hrsa.gov/data/download>). County-level linked measures were calculated as number per 1,000 population and included number of hospitals, number of primary care providers, and number of obstetricians-gynecologists (Ob-Gyns). Hospital referral region (HRR)-level availability metrics derived from Medicare and Medicaid data from the Dartmouth Atlas Project (<https://atlasdata.dartmouth.edu/downloads>) were linked using patient zip code and year of diagnosis. HRR data captures the characteristics of the regional markets for tertiary healthcare systems. HRR-level availability metrics of interest for the patient’s year of diagnosis were acute care beds available per 1K population, physicians per 100K population, primary care physicians per 100K population, Hematologists/Oncologists per 100K population, Ob-Gyns per 100K women aged 15-44, percentage of Medicare beneficiaries that died, percentage of beneficiaries seeing a primary care physician (PCP) that year, discharges for ambulatory sensitive conditions per 1K population, hospital discharge 30 day readmission rates, and hospital discharge 30 day return to emergency room (ER) rates. For metrics without data available for each calendar year, the information was imputed from the most proximate year available to the patient’s diagnosis within five years. The National Cancer Institute (NCI) hospital file was used to determine facility-associated availability metrics including the hospital’s ownership, affiliation with a medical school, NCI Cancer Center designation critical access status, and number of beds in the year of the patient’s cancer diagnosis. If the hospital’s information was missing in a calendar year, the information was imputed as the highest availability value for the hospital recorded in the study time period.

*Factor analysis and creation of HCA factor scores*

We used the Penchansky and Thomas framework of healthcare access to guide our selection of variables representing the hypothesized latent constructs of healthcare access (affordability, availability, and accessibility) to include in our analysis using a two-stage confirmatory factor analysis approach. First, factor analysis was conducted for each *a priori* grouping of HCA dimension measures (Affordability, Availability, and Accessibility), then variables with significant loadings in the three preliminary models were carried forward into one final combined model. Two variables measuring number of specialists available (gynecologic oncologists and OB-GYNS) had high correlation efficiency, thus the gynecologic oncologist variable was excluded. A total of 18 HCA dimension measures were carried over and loaded into the second stage factor analysis. There was a clear separation for each of the three hypothesized factors (representing Affordability, Availability, and Accessibility) on the factor analysis scree plot, and each factor captured the majority of measures for a hypothesized HCA domain. We next conducted reliability tests and assessed model fit for these selected factors. The reliability test results and model fit index are presented in Supplementary Table 2. Based on the reliability tests, we adjusted our final factor model by excluding the number of hospitals per 1K county population variable, which resulted in improved reliability metrics for the accessibility domain. The final factor model comprised a total of 13 variables loading onto the 3 factors, with close to 89% of the sample variance was explained. We also conducted exploratory factor analysis to agnostically determine factor structure for HCA domains. However, the 3-factor model did not demonstrate a simple and clear structure with respect to which variables loaded together on each factor, and factors 2 and 3 had low reliability scores when assessed using Cronbach’s alpha coefficient. Therefore, to improve interpretability and reliability of the factor scores, we relied on the confirmatory factor analysis approach.

Estimated factor scores for each HCA domain were created using PROC FACTOR to generate a linear composite of optimally weighted variables under analysis. To test heterogeneity of the associations by patient race and ethnicity, values of factor scores were stratified by race and ethnicity. Factor weighted sum scores were compared for each factor across patient race and ethnicity. Scores were centered at zero, with values ranging from approximately -3 to 4, with negative values representing the lowest scores for the dimension (i.e. low affordability), and positive scores representing higher scores for the dimension. Factor analyses were conducted using SAS version 9.4 (2013, SAS Institute, Cary, NC).
